# The use of HEAVEN criteria to predict difficult laryngeal view and intubation failure with direct and video laryngoscopy

**DOI:** 10.1186/s13049-020-00806-w

**Published:** 2020-11-11

**Authors:** Ryan D. Henschell, Paul K. Miller

**Affiliations:** Rocky Vista University - College of Osteopathic Medicine, 8401 S Chambers Rd, Parker, CO 80134 USA

**To the Editor**

We read with interest the paper by Nausheen et al. [1] in which the authors report that the HEAVEN criteria [2] predict laryngoscopic view and intubation success for direct and video laryngoscopy. They conducted a retrospective analysis of 5137 patients who underwent rapid sequence induction (RSI) of anesthesia prior to intubation. In their study each of the HEAVEN criteria was associated with lower intubation success rates with and without desaturation, and the total number of HEAVEN criteria present was inversely proportional to intubation success with and without desaturation.

Nausheen et al. report that as the number of HEAVEN criteria increases, the difficulty of laryngoscopy increases. But we are perplexed as to why the presence of three criteria confers less difficulty than the presence of two.

The authors also report that air medical crews applied the HEAVEN screening tool prior to RSI, and if one or more criteria were present, the crews were encouraged to use alternative (intubation) strategies. We wonder why strategic positioning of the patients prior to attempted laryngoscopy/intubation was not included. Optimal positioning of an obese patient has been shown to improve the ease of laryngoscopy and intubation [3].

Additionally, it strikes us as odd that, in Fig. 1, the physiologic phenomenon of “exsanguination” negatively impacts the direct laryngoscopic (DL) and video laryngoscopic (VL) views of the airway more than the physical phenomena of extremes of size, vomit/blood/fluid in the pharynx and neck mobility issues, as well as VL in patients with anatomic challenges. Similarly strange is that direct and video laryngoscopy in hypoxemic (Sp0_2_ < 93%) patients are as difficult as in patients with “clinically significant” vomit/blood/fluid in their mouths and throats.

Table 2 reveals, that in spite of the difficult airway views noted in patients with hypoxemia or exsanguination, there was no statistically significant association of such views with failure at first intubation attempt utilizing either DL or VL. With respect to failure of first intubation attempt without desaturation, there was no statistically significant correlation of VL in the setting of exsanguination.

It is not surprising that four of the six HEAVEN criteria (extremes of size, anatomic challenges, vomit/blood/fluid, neck mobility issues) are associated with difficult laryngoscopy and intubation. But it appears that the inclusion of two purely physiologic parameters (exsanguination, hypoxemia) produces curious results.

We invite the authors to respond to these comments.

## Data Availability

Data sharing not applicable to this article as no datasets were generated or analyzed during the current work.
